# Synthesis and in vitro PDT evaluation of red emission polymer dots (R-CPDs) and pyropheophorbide-α conjugates

**DOI:** 10.1038/s41598-021-89081-y

**Published:** 2021-05-11

**Authors:** Faiza Sajjad, Yi-Jia Yan, Davor Margetić, Zhi-Long Chen

**Affiliations:** 1 Department of Pharmaceutical Science & Technology, College of Chemistry and Biology, DongHua University, Shanghai, 201620 China; 2 Shanghai Xianhui Pharmaceutical Co., Ltd, Shanghai, 200433 China; 3 Division of Organic Chemistry and Biochemistry, RuđerBošković Institute, Bijenička c. 54, 10000 Zagreb, Croatia

**Keywords:** Cancer, Medical research, Chemistry, Nanoscience and technology

## Abstract

Carbon based polymer dots have piqued attention of researchers because of excellent biocompatibility, and good solubility. Most of the p-dots are able to generate ROS which is effective for photodynamic therapy for the treatment of cancer while some photosensitizers such as porphyrins possess some drawbacks such as hydrophobicity, and dark toxicity. Therefore in this study we conjugated red emission carbon based polymer with pyropheophorbide-α through amide condensation and π–π stacking. One pot synthesis of the conjugate was successfully achieved. Their photophysiological properties were studied and structures were characterized by FT-IR, TEM and ^1^HNMR. pH- sensitivity of the conjugates was confirmed using fluorescence and UV–vis spectroscopy. Photo toxicity and dark toxicity of the prepared conjugates were evaluated in human esophageal cancer cell line (Eca-109). Hemocompatibility of the synthesized conjugates was evaluated and proved that the conjugates are safe to use for the treatment of tumor. Our results showed the PS doped p-dots had less dark toxicity and increased light toxicity as well as ROS generation was high as compared to precursor drug. Therefore, incorporation of p-dots to porphyrin improved biocompatibility and enhanced the photodynamic effect.

## Introduction

Carbon-based fluorescence nanomaterials, such as carbon dots (CDs) and polymer dots (PDs), have stirred much attention, because of their enticing properties, e.g., high fluorescence^1–4^, excellent stability^5,6^ and good biocompatibility^7–10^. Although carbon dots and polymer dots both possess quasi-spherical shape but CDs possess inorganic carbon-based cores, while PDs have organic skeleton. CDs and PDs have been widely applied in the fields of sensing^11–15^, bioimaging^16–19^, and photodynamic/photothermal therapy (PDT/PTT)^5,8,10,19,20^ as alternatives for commercial semiconductor quantum dots and organic dyes. Different methods have been developed to produce CDs, including laser irradiation^21,22^, hydrothermal synthesis^23,24^, electrochemical etching^25^, and ultrasound and microwave-assisted syntheses^26,27^. These methods suffer from various limitations, such as high energy consumption and complex experiments. Moreover a high energy input would produce a large number of by-products^26–29^, which further require additional techniques for purification^30^. These problems limit the synthesis of CDs on a large scale. Additionally, their short-wavelength emission (400–550 nm) and poor water solubility further limit the use of semiconductor quantum dots and organic dyes in the fields of bioimaging and nanomedicine^31–33^.

Polymer dots (Pdots, PDs) have been developed as a brightly emissive nanoprobe used for bioimaging, diagnosis of the disease and drug delivery (therapy). Pdots are suitable for these different applications because of their excellent photophysical properties, high extinction coefficients, extraordinary particle brightness, and excellent photostability. Moreover, Pdots also possess good biocompatibility, tunable optical properties and surface properties and colloidal dimensions^34^. Most importantly, the carbon polymer dots (CPDs) have some drawbacks such as, short-wavelength emission, thus limit their application in bioimaging. Porphyrins belong to the family of tetrapyrrole has been extensively investigated as photosensitizers (PSs) for the treatment of tumors using PDT. Though porphyrin-based molecules are the most commonly used PSs for PDT, many of them possess some drawbacks such as prolonged photosensitivity, poor solubility, dark toxicity, effective PDT and inadequate selectivity yield. To overcome these shortcomings, the incorporation of nanomaterials to porphyrins through self-assembly or covalent linkage is one of the effective methods^35,36^. Red emission carbon based polymer dots (R-CPDs) synthesized by Xia^37^ and his colleagues showed excellent resistance to photo bleaching, low cytotoxicity and pH sensitivity. Pyropheophorbide-α is a (PPa) chlorophyll derivative with high ROS generation but owns certain drawbacks such as poor solubility, dark toxicity and low biocompatibility. One approach to overcome those problems is to functionalize the PS with compounds that have good compatibility with the tissue. So, to develop, compare and evaluate the efficient method to improve the overall efficacy of the photosensitizers used for PDT, in this study we synthesize R-CPDs with some modification and then conjugated R-CPDs to PPa through covalent and π–π stacking. We also developed a facile one pot synthesis method for the incorporated conjugates which not only reduces the synthesis time and work but also decrease the toxicity of the PSs. Their photo-physiological properties and in vitro evaluation was performed to validate its potential use in biomedical application. The as-synthesized R-CPD conjugates not only accentuate the importance of functionalization of the nanomaterials but also provide a feasible approach to improve the therapeutic efficacy of the PSs.

## Material and methods

PPa was acquired from Shanghai Xianhui Pharmaceutical Co., Ltd. All other chemicals and reagents were purchased from Sinopharm Chemical Reagent Co., Ltd and used without further purification. The blood was acquired from Zhenhu Medical Technology Co., Ltd. All solutions were freshly prepared with deionized water and all reactions were protected from sunlight. ^1^H NMR spectra were recorded on a Bruker AMX-400 and chemical shifts δ (ppm) were referenced to TMS. UV–Vis absorption spectrum was recorded on an ultraviolet visible spectrophotometer (Model V-530, Japan). Fluorescence spectra were measured on a fluorescence spectrophotometer (FluoroMax-4, France). FTIR of the PD-hybrids was recorded on Perkin Elmer, spectrum-Two FT-IR and TEM images were taken from JEM-2100. Dynamic light scattering (DLS) experiments were done on a dynamic light scattering instrument BI-200SM.

### Synthesis of R-CPDs

R-CPDs were synthesized according to the literature with some modifications^37^. Briefly, 10 mM of p-phenylenediamine (pPD) and 100 mM FeCl_3_ was dissolved in 15 mL ultrapure water. The reaction mixture was heated at 120 °C for 24 h in the air. The mixture was then filtered with a 0.22 µm polyethersulfone membrane, dialyzed and the retentate/filtrate was lyophilized to obtain a powder.

### Self-assembly of PDs to PPa (PPa-PD1)

**Figure 1:**
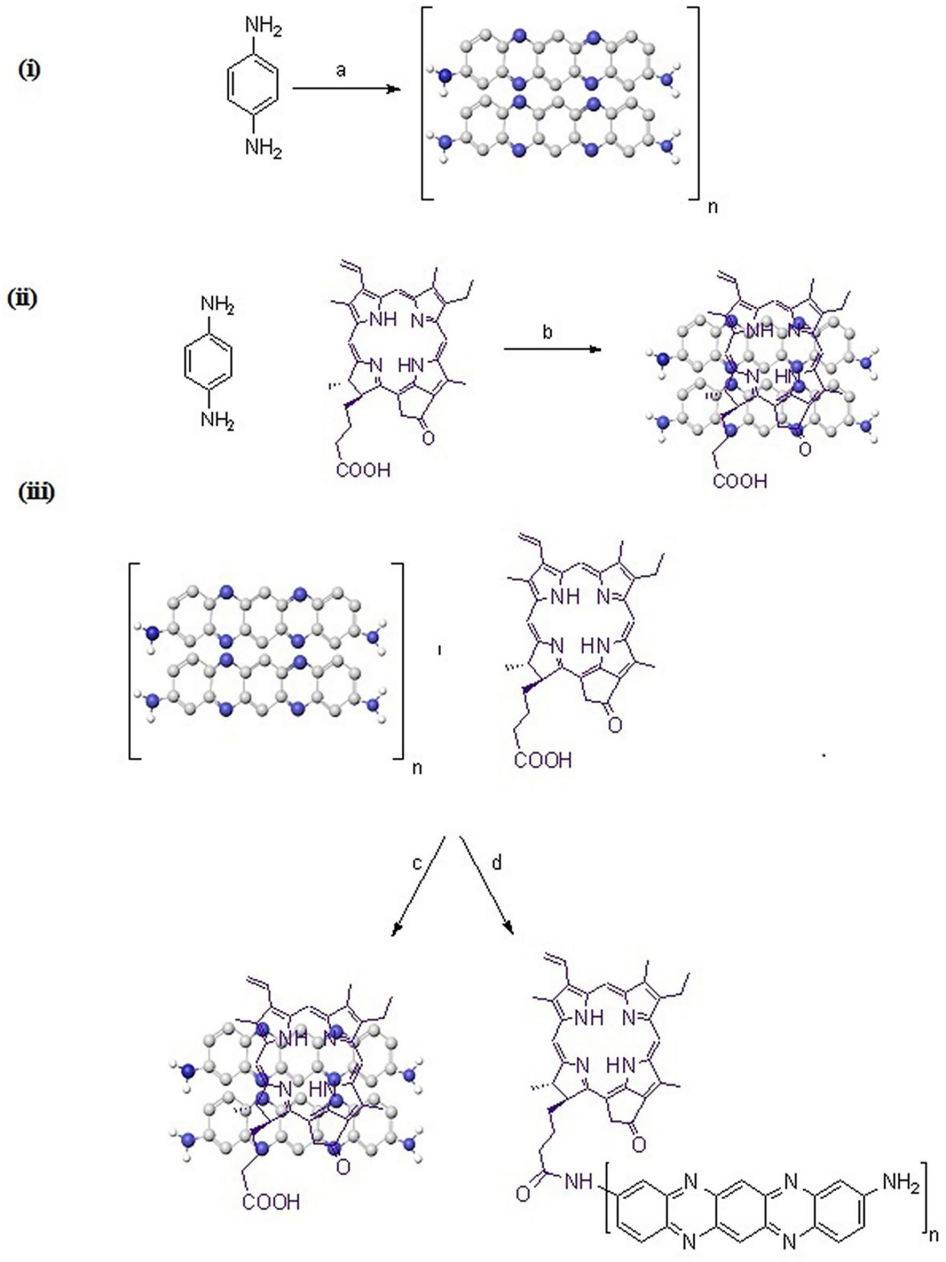
(i) Synthesis of R-CPDs and conjugates, (**a**) FeCl2, 80 °C, (ii) one pot synthesis of PPa-PD3, 120 °C, (iii) Conjugation of PPa-PD1 and PPa-PD2, (**c**) sonication of 48 h, (**d**) DIPEA, HBTU.

The non-covalent coordination (π–π stacking) of PDs with pyropheophorbide-α was achieved following a method employed before.^38^. The PDs (30 mg in 1 mL water) and pyropheophorbide-α (36.0 mg, 0.065 mol) were dispersed in 2.0 mL dry DMF and sonicated for 4 h. The mixture was then stirred at room temperature for 48 h followed by successive washing with ethanol, and deionized water by centrifugation. The product was finally washed with diethyl ether and dialyzed with DI water (Fig. 1i).

### Covalent bonding between PPa and PDs (PPa-PD2)

In the synthesis of R-CPD-PPa conjugate (PPa-PD2), PPa (24.2 mg, 0.043 mol) and HBTU (14.0 mg, 1.2 M) were dispersed in 2.0 mL dry DMF, 2.5 M of DIPEA, and 30 mg of R-CPDs in water was added and the mixture was stirred at room temperature for 12 h. The product was then collected and successively washed with deionized water, ethanol by centrifugation and dialyzed with DI water to remove the residual byproduct (Fig. 1ii).

### One-pot synthesis PPa.PD3

One-pot synthesis was successfully achieved by dissolving 10 mM of p-phenylenediamine, 100 mM FeCl_3_, and pyropheophorbide-α (2 mg, 0.0036 mol) in ultrapure water. The reaction was then stirred for 24 h at 120 °C. The mixture was then centrifuged several times to separate nano-conjugates from unreacted entities using ethanol. The product was filtered and dialyzed for two days (Fig. 1iii).

### Absorption and emission spectra

Absorption spectra were recorded using ultraviolet–visible spectrophotometer. Spectra were collected from 300 to 800 nm. Emission spectra were recorded using fluorescence spectrometer from 300 to 800 nm wavelength in 1 nm steps. Conjugates were dissolved in different solvents (DMSO, MeOH and DMF) at room temperature using quartz cuvettes.

### Photo-bleaching assay

The photo stability of the synthesized conjugated was tested in DMSO. The absorption spectra of the nano-hybrids were measured at final concentrations of 10 µM using 1 cm quartz cuvettes. Then, the solutions were irradiated with a semiconductor laser unit (PDT—670 nm), and the output power and discontinuous irradiation time were fixed to 5 mW/cm^2^ and 120 min. After irradiation, the absorbance of the conjugated drugs was measured again. The degree of photo-bleaching was calculated according to the equation: percent (%) = A_t_/A_0_ × 100, where A_0_ and A_t_ represent the absorbance before and after irradiation, respectively, and t is the irradiation time (10–120 min).

### Reactive oxygen species detection

The DPBF and each conjugate (2 µM) was mixed at a 30 to 1 ratio in quartz cuvettes, which were irradiated every 5 s in the presence of oxygen using a 5 mW/cm^2^ Nd: YAG laser (670 nm) as the light source. The absorption spectra were measured by ultraviolet–visible spectrophotometer. Afterwards, the relative consumption of DPBF indicates the amount of singlet oxygen present. The rate of singlet oxygen generation calculated by the following equation described by

$${\text{ln}}\;\left( {\left[ {{\text{DPBF}}} \right]{\text{t}}/\left[ {{\text{DPBF}}} \right]0} \right) = - {\text{kt}}$$

where [DPBF]_t_ and [DPBF]_0_ are the concentrations of DPBF after and prior irradiation, respectively. Values of k are the rate of singlet oxygen generation and t is the time duration of irradiation^39^.

### Cell culture

Human esophageal cancer cell line (Eca-109 cells) was purchased from Institute of Biochemistry and Cell Biology, CAS. The cells were maintained in RPMI medium, supplemented with 10% FBS, 100 IU/mL penicillin, and 100 mg/mL streptomycin at 37 °C in a humidified atmosphere of 5% CO_2_.

### Cellular uptake of conjugates

Eca-109 cells were cultured and grown in 12-well plates at 5 × 10^4^ cells/well. 24 h later, the medium was replaced with fresh medium containing 2 μM compounds and incubated for an additional 24 h. The cellular uptake of the compound was determined by a fluorescence spectrophotometer according to the method previously reported^40^.

Cellular uptake of the conjugates was observed using a fluorescence microscope. Cells were cultured over coverslips. After 24 h, 2 µM of each conjugate was added and incubated overnight and then observed under a microscope^41^.

### Cytotoxicity of conjugates

The conjugates in PBS were filtered and sterilized. A semiconductor laser was applied as a light source in PDT. Cells were cultured in 96 well plate at 5 × 10^4^ cells /100 μL per well for 24 h, treated with 0, 1, 2, 3, 4, 5 µg/mL of PD-conjugates for dark toxicity and 0, 0.1, 0.25, 0.5, 0.75, 1 µg/mL for phototoxicity. Light exposure was regulated by irradiation time, with the intensity of 2 J/cm^2^, obtained with illumination times for 1 min 40 s. Afterward 20 μL MTT was added per well for 4 h, and absorbance was measured with a microplate reader. The data was presented as mean ± standard deviation (SD)^42^. The cell viability was calculated according to the following formulation: cell viability (%) = OD experiment/OD control × 100%. All experiments were carried out in triplicate. Cytotoxicity of the conjugates was also confirmed through florescence microscope using live and dead staining.

### Hemolytic assay

To determine the effect of CD-conjugates on blood, the hemolytic assay was performed. Mouse blood was centrifuged (16,000 rpm) for 5 min to collect erythrocyte. The obtained erythrocytes were washed with PBS three times and diluted. After that 500 μL of diluted blood was added in tubes followed by the addition of 500 μL of each conjugate, distilled water, PBS and incubated for 1 h at 37 °C. After incubation, each tube was centrifuged for 5 min at 16,000 rpm. The absorbance of collected supernatants was recorded at 545 nm with a microplate reader. PBS served as negative control while distilled water as a positive control. Hemolysis ratio was calculated using following formula^43^;

$${\text{Hemolysis}}\;{\text{ratio}}\;\left( {{\text{HR}}} \right) = \left[ {\left( {{\text{Ap}} - {\text{Ab}}} \right)/\left( {{\text{At}} - {\text{Ab}}} \right)} \right] \times {1}00\%$$

where Ap was the absorbance value of supernatant from each group, At was the absorbance value of the Triton X-100 positive control and Ab was the absorbance value of DPBS. Each group contains three repeats.

### Whole blood clotting

The effect of the conjugated drugs on blood clotting was evaluated. A volume of 50 μL of recalcified whole-blood solution (0.2 M CaCl_2_, 10 mM in the blood) was added to drug (37 °C) in polypropylene tubes. Then, the tube was incubated at 37 °C for 30 s, 60 s, 90 s, and 120 s, respectively. Blood was used as a control group. After pre-set time 1 mL of distilled water was added gently without disturbing the clot. The absorbance of the supernatant was recorded at 540 nm by using a microplate reader. Three replicates were performed.

### pH sensitive response

The pH sensitivity of the synthesized nano-hybrids in different solutions (pH 4–8) were estimated. About 10 μM concentration of each conjugate was dissolved in different pH solutions and emission spectra were taken through fluorescence spectrophotometer^37^. The pH sensitivity was also tested using UV–vis spectrophotometer following the method reported previously^44^.

### Statistical analysis

Graphs were created by origin 8.0 (Graph Software, USA). All results are presented as mean ± SD. Statistical significance was determined by unpaired two-tailed t tests or two-way analysis of variance. A P value of < 0.01 and < 0.05 was considered statistically significant.

## Results and discussion

### Preparation of R-CPDs and PPa-PD conjugates

The R-CPDs were synthesized using FeCl_3_ as a catalyst to modulate polymerization of p-phenylenediamine in ultrapure water. The reaction mixture was stirred for 24 h. The filtrate was dried to obtain dark color powder. The π–π stacking of PDs with pyropheophorbide-α was achieved by dissolving PDs and pyropheophorbide-α in dry DMF. The mixture was sonicated for 4 h and then the reaction was continued for 48–72 h. The nano-hybrid was then washed and centrifuged with ethanol, and deionized water.

For the covalent linkage between PPa and PDs, PPa (24.2 mg), HBTU and DIPEA were dispersed in 2.0 ml dry DMF and, stirred for 2 h. Then 30 mg of R-CPDs solution was added and the mixture was stirred at room temperature for 12–24 h. The dark color product was collected after washing with ethanol. One pot approach was also used for the synthesis of the conjugated compound. PPa was dissolved in water, following the addition of pPD and FeCl_3_ and let it react for 24 h. The unreacted species were then separated by centrifugation. The conjugated drug was filtered, and dialyzed to get pure product.

### Structural characterization

**Figure 2:**
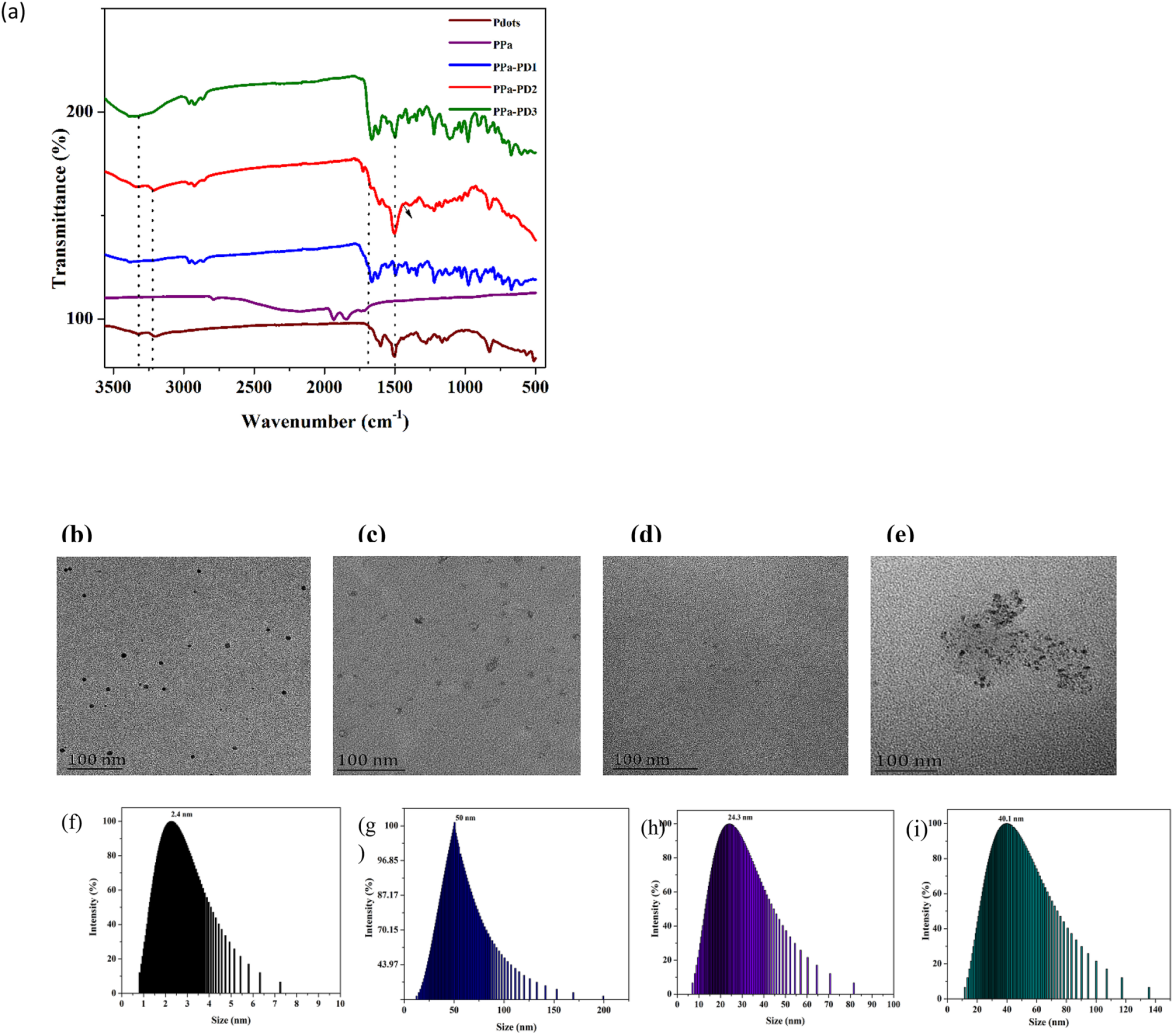
(**a**) FTIR of R-CPDs and conjugates. TEM images of (**b**) R-CPDs, (**c**) PPa-PD1, (**d**) PPa-PD2, (**e**) PPa-PD3. DLS of (**e**) R-CPDs, (**f**) PPa-PD1, (**g**) PPa-PD2, (**i**) PPa-PD3.

The synthesized R-CPDs and conjugates were characterized by FT-IR, TEM and ^1^HNMR. Figure 2a illustrates the FT-IR spectra of the hybrid compounds. The peak at 3200 cm^−1^ refers to the N–H group while the peak around 1600 cm^−1^ predicts the presence of C = N/C = O. Retention of the bands in the region around 3000–3500 cm^−1^ suggests the presence of amide and hydroxyl groups in the prepared conjugates. The peak around 1200–1400 cm^−1^ refers to the presence of aliphatic compounds. The appearance of a peak in PPa-PD2 at 1433 cm^−1^ suggests the formation of an amide bond. The peak around 1600 cm^−1^ refers to the C=C/C=N bond. The ^1^HNMR of PPa and PD conjugates confirmed the structures of nano-hybrids. The incorporated drugs were further confirmed by ^1^HNMR spectroscopy (Fig S1a-e). The peak around 7.4 ppm (PPa-PD2) refers to the formation of an amide bond. The spectra showed peaks at 7–9 ppm which indicates the aromatic (sp^2^) hydrogen atoms of the precursor drug. To further strengthen the structural studies of the PD-conjugates transmission electron microscopy was done. The morphology and size of the nano-hybrids is shown in Fig. 2b–e. The sizes of the conjugates were confirmed by Dynamic light scattering (DLS). An increase in the distribution frequency of the diameter of each integrated compound showed the successful incorporation of PDs to the porphyrin molecule. The huge increase in size after conjugation suggests the aggregation of porphyrins as, porphyrins tend to aggregate when interacted with nanoparticles due to the adsorption of porphyrins into the nanoparticles (Fig. 2f–i). In porphyrins, aggregation by π–π stacking is common^38^. It was observed that the size of PPa-PD3 was smaller than PPa-PD1, which suggests that the temperature might affect the aggregation of the porphyrins when interacted with nanoparticles.

### Photophysical studies

**Figure 3:**
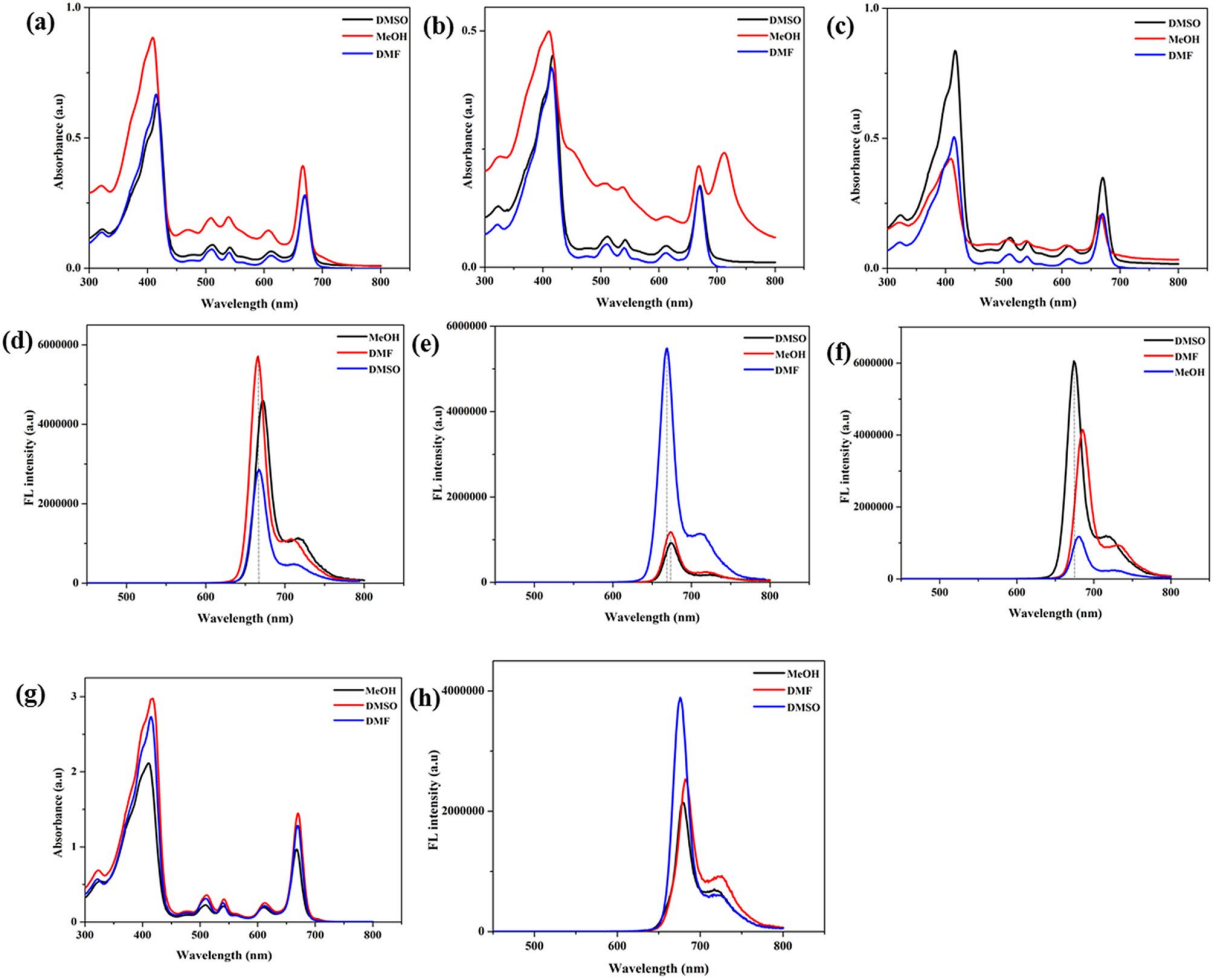
Absorbance of R-CPD conjugates (**a**) PPa-PD1, (**b**) PPa-PD2, (**c**) PPa-PD3, (**d**) Flourescence spectra of PPa-PD1, (**e**) PPa-PD2, (**f**) PPa-PD3, (**g**) PPa absorption spectra, (**h**) PPa fluorescence spectra.

Absorption and emission spectra of photo luminescent compounds are important for diagnosis and treatment. Figure 3a–c illustrated the absorption spectra of the PD-nanocomposites in different solvents. The synthesized hybrids showed a broad absorption from 400 to 670 nm. The absorbance of the conjugates had a shift of 2 nm from precursor drug (Fig. 3g) which is 668 nm in different solvents while for PPa-PD2 the absorbance peak at 713 nm was observed which suggest the successful conjugation. The shift in the peak might be because of the high polarity of methanol. Porphyrins as well as R-CPDs are known for their photoluminescence. The fluorescence emission spectra of conjugates synthesized following different mechanisms were depicted in Fig. 3d–f. Some organic drugs possess solvatochromic effect, different fluorescence in different solvents^45,46^. These results depicted the solvent-dependent fluorescence emission of PPa-PD hybrids. Fluorescence peak was observed at 675 nm in DMSO while in the PPa-PD3 peak was shifted to 685 nm which was at 681 nm in PPa (Fig. 3h) when dissolved in DMF.

### Photostability

**Figure 4:**
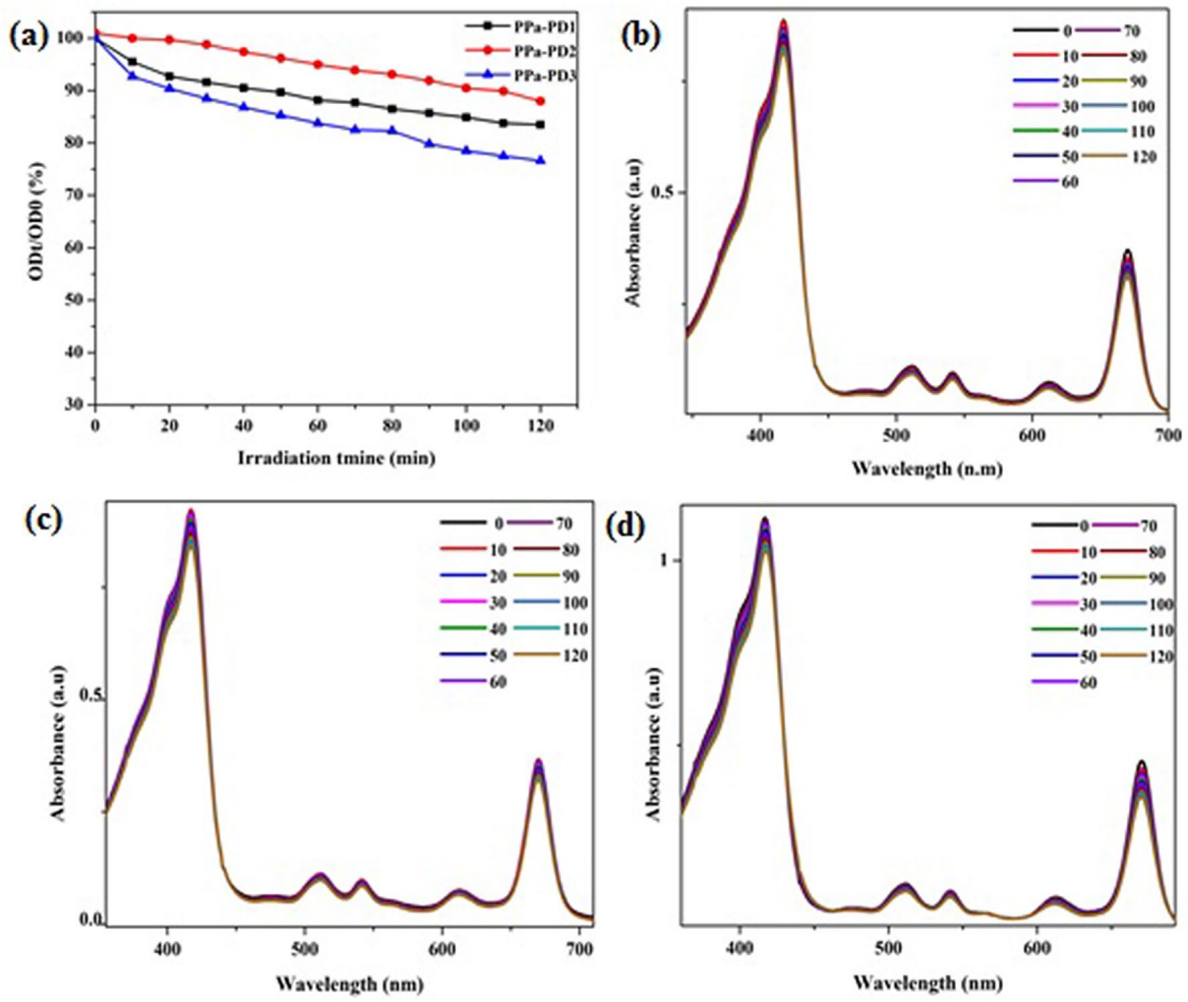
(**a**) Photostability of the conjugates in DMSO, (**b**) PPa-PD1, (**c**) PPa-PD2, (**d**) PPa-PD3.

One of the concerning issues with the photosensitive drugs is stability. The rate of photostability plays an important role in photodynamic therapy^47^. The photostability of the drugs was evaluated and, the OD values of maximum absorption showed no significant change with the irradiation time after 2 h of irradiation indicated that the conjugated PDs were stable and, the structure of compounds did not change after 120 min of laser treatment (Fig. 4). One eminent advantage of the covalently PPa-incorporated Pdots is their excellent stability that solves the photosensitizer leaching problem.

### ROS generation

**Figure 5:**
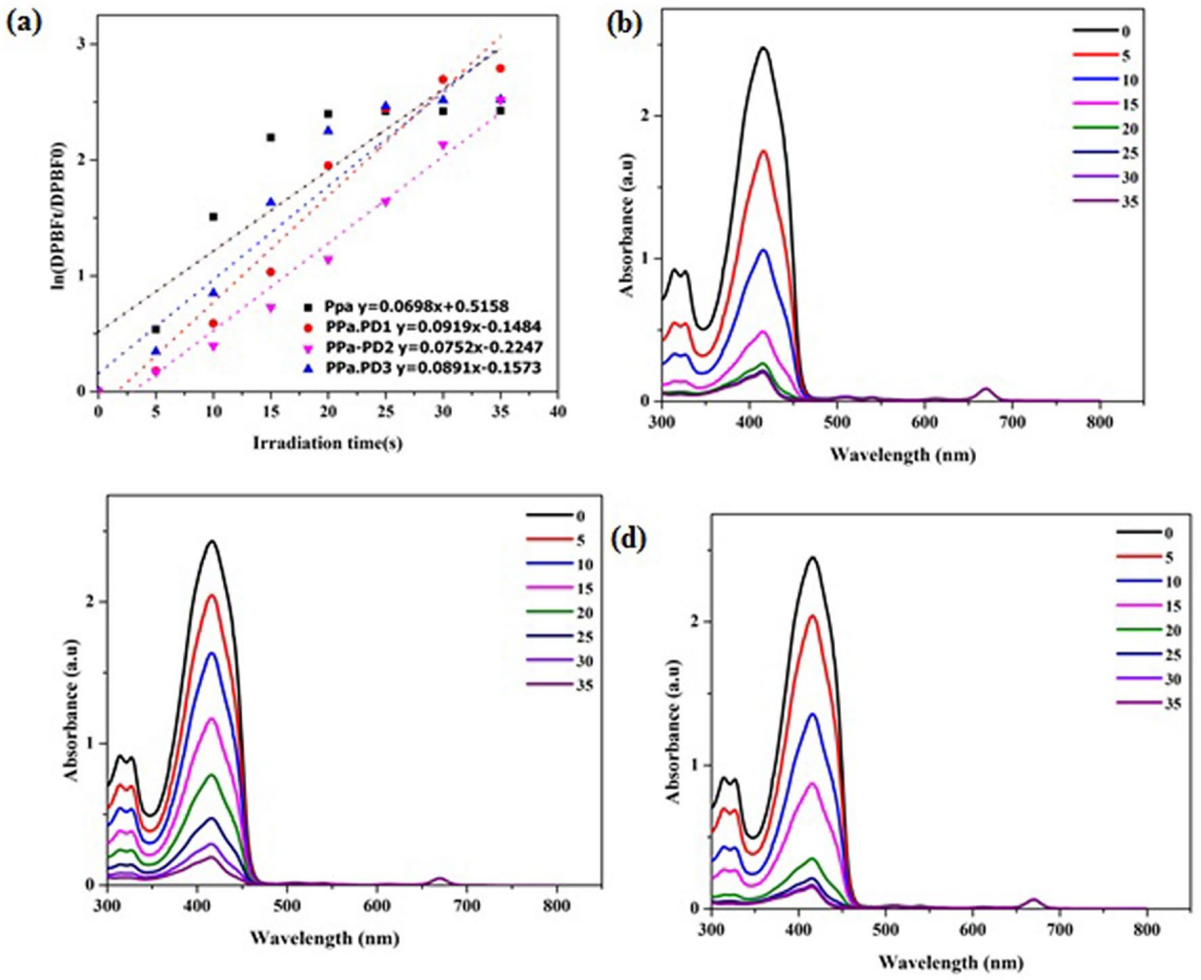
(**a**) ROS generation in nano-hybrids. Photodecomposition of DPBF in (**b**) PPa-PD1, (**c**) PPa-PD2, (**d**) PPa-PD3.

Singlet oxygen generation by photosensitizers is another important factor affecting PDT. Different scavengers of singlet oxygen are present nowadays. We used 1,3-diphenylisobenzofuran (DPBF) to determine the rate of ROS generation^48^. Upon laser irradiation, the excited nano-hybrids generated singlet oxygen. The singlet oxygen produced as a result of irradiation then induced tumor destruction. Our studies showed that the incorporation of PDs to PPa increased the singlet oxygen as compared to the precursor compound. The decrease in absorbance of DPBF was remarkable in each hybrid drug. Each PD hybrid showed fast bleaching of DPBF (Fig. 5).

### Dark and light toxicity

**Figure 6:**
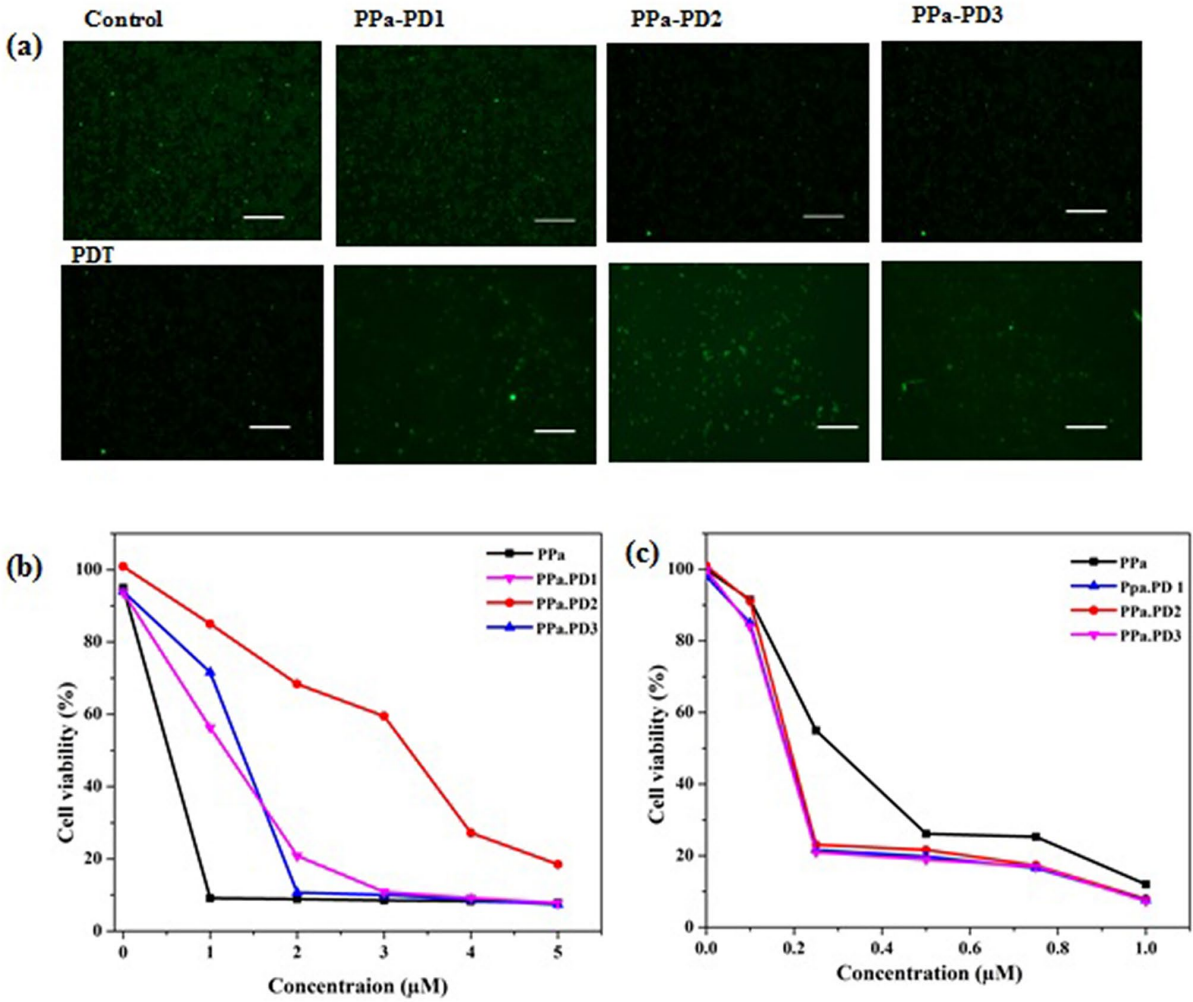
(**a**) Cell viability of Eca-109 after PDT. (**b**) MTT assay dark toxicity, (**c**) Phototoxicity. Scale bar 200 μm.

The increased ^1^O_2_ yield and good photostability of PPa-Pdots make them a promising candidate for photodynamic cancer treatment. The dark and light toxic effect of the conjugated drugs was studied using MTT assay and fluorescence microscopy. We used cellular staining to investigate the photodynamic toxicity induced by the PPa-PDs. The staining of the cells showed apparent cell death after treatment with Pdots and light irradiation as compared to the control groups in the absence of Pdots incubation or light irradiation (Fig. 6a). The results of the MTT assay further confirmed the PDT induced toxicity of the conjugates. As illustrated in Fig. 6b,c the cellular viabilities decreased apparently as the Pdot concentration increased. The cell viability showed a negligible decreased after incubation with Pdots at high concentration (5 μg/mL), indicating the good biocompatibility of the Pdots in the absence of light. The low dark toxicity of the Pdots is one of the important characteristics of their practical applications. The cell staining results together with the cell viability studies indicated that PPa-Pdots could function as a potent nanoparticle photosensitizer for cancer cell damage due to the efficient ROS generation.

### Cellular uptake

**Figure 7:**
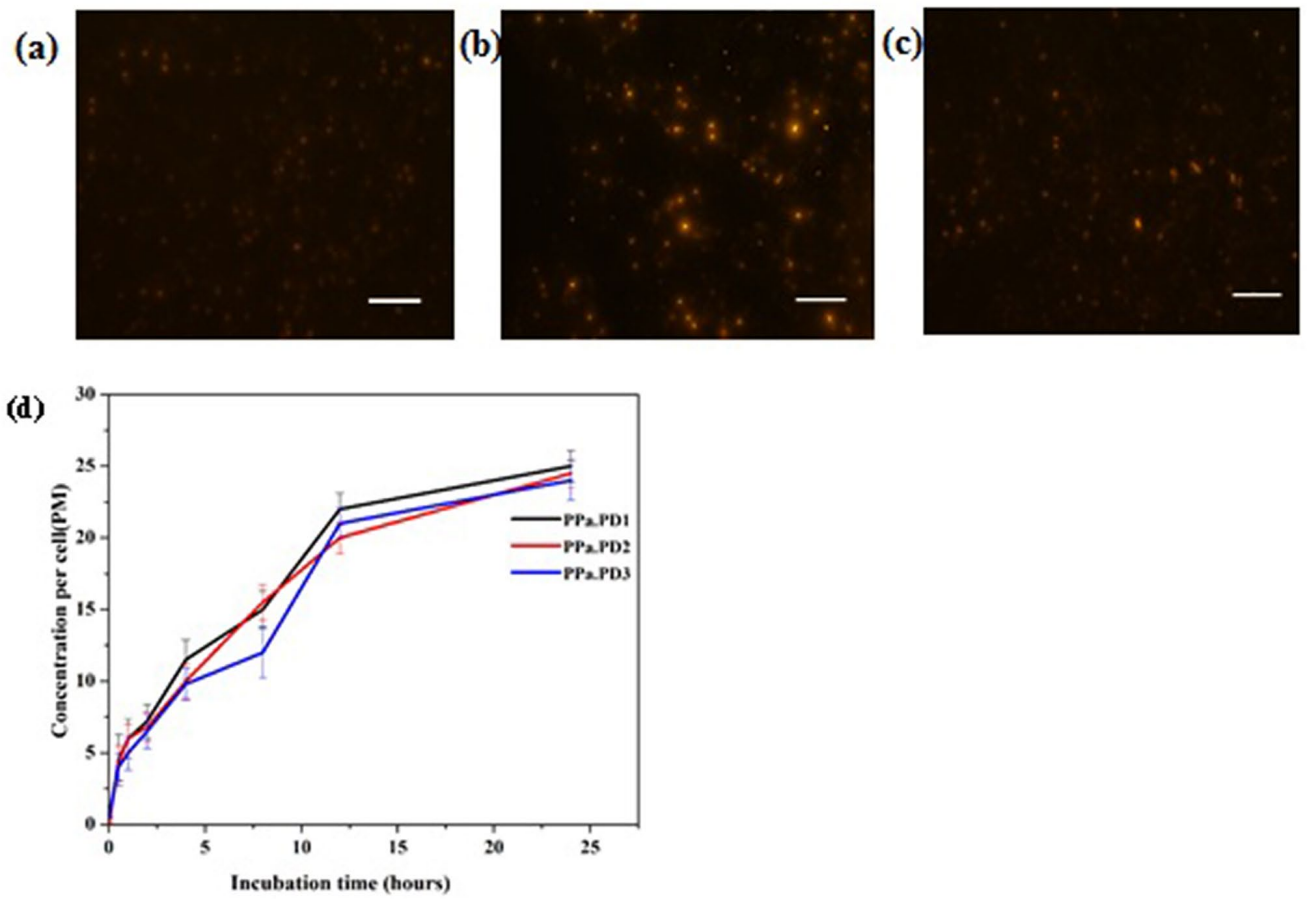
PD conjugates uptake by Eca-109 (**a**) PPa-PD1, (**b**) PPa-PD2, (**c**) PPa-PD3 (d) Cellular uptake. Scale bar 200nm.

To investigate the cellular uptake of pdot hybrids, the fluorescence imaging was performed on Eca-109. After incubation for 24 h, Eca-109 cells showed fluorescence when observed under a fluorescence microscope indicating the accumulation of the drug in cells (Fig. 7a–c). The cellular uptake was further confirmed by fluorescence analysis as shown in Fig. 7d. After 24 h of incubation, maximum absorption was observed. So, the synthesized nano-hybrids can be absorbed by the cells and produced efficient PDT actions.

### Hemocompatiblity

In vitro hemolysis assay is a universal method to evaluate the hemocompatibility of materials^49^. The hemolysis ratios of the conjugates were tested. The macroscopical color of centrifugally obtained supernatants for drug groups, and control groups was shown in Fig S2a. The color of the supernatant obtained depicted the hemolytic ratio of the erythrocytes when treated with 5 µg of conjugates and their precursors. Drug groups presented light yellow similar to the PBS control group, while the positive group was bright red. The quantitative data of the hemolysis ratio was shown in Table 1. PPa-PD conjugates showed a low hemolysis ratio as compared to the precursor molecule revealing its best hemocompatibility. If the hemolysis rate is below 5%, medical materials will be considered as non-hemolysis according to national biological safety^50^. The results of the samples were all less than 5%, conforming to the national biological material hemolysis rate security specified requirements. The blood clotting was tested using each conjugate. Our results showed that during first 30 s OD of the control was higher than the drug groups but at the end of 120 s the OD of the control and hybrid drug was the same while its higher in case of precursor molecule, suggesting that the conjugation of PDs to PS help to maintain hemostasis of the body fluids. The OD of the PPa-PD1 and PPa-PD3 were much lower after 120 s suggesting that the drugs were hemocompatible and did not affect the normal hemostatis of the body (Fig.S2b). The results of the hemolysis ratio and clotting test suggested that the incorporation of the p-dots with the organic drugs could improve hemocompatibility.

**Table 1:** Hemolysis caused by different conjugates (5 µg).

Samples	Optical density at 545 nm	Hemolysis ratio (%)
PBS	0.0710 ± 0.0113	0
Water	1.5682 ± 0.0197	100
PPa	0.0928 ± 0. 0169	1.47
PPa-PD1	0.0859 ± 0.01202	0.99
PPa-PD2	0.0877 ± 0.01463	1.11
PPa-PD3	0.0912 ± 0.00155	1.34

Standard deviation is calculated from three samples.

### pH sensitivity of R-CPD conjugates

**Figure 8:**
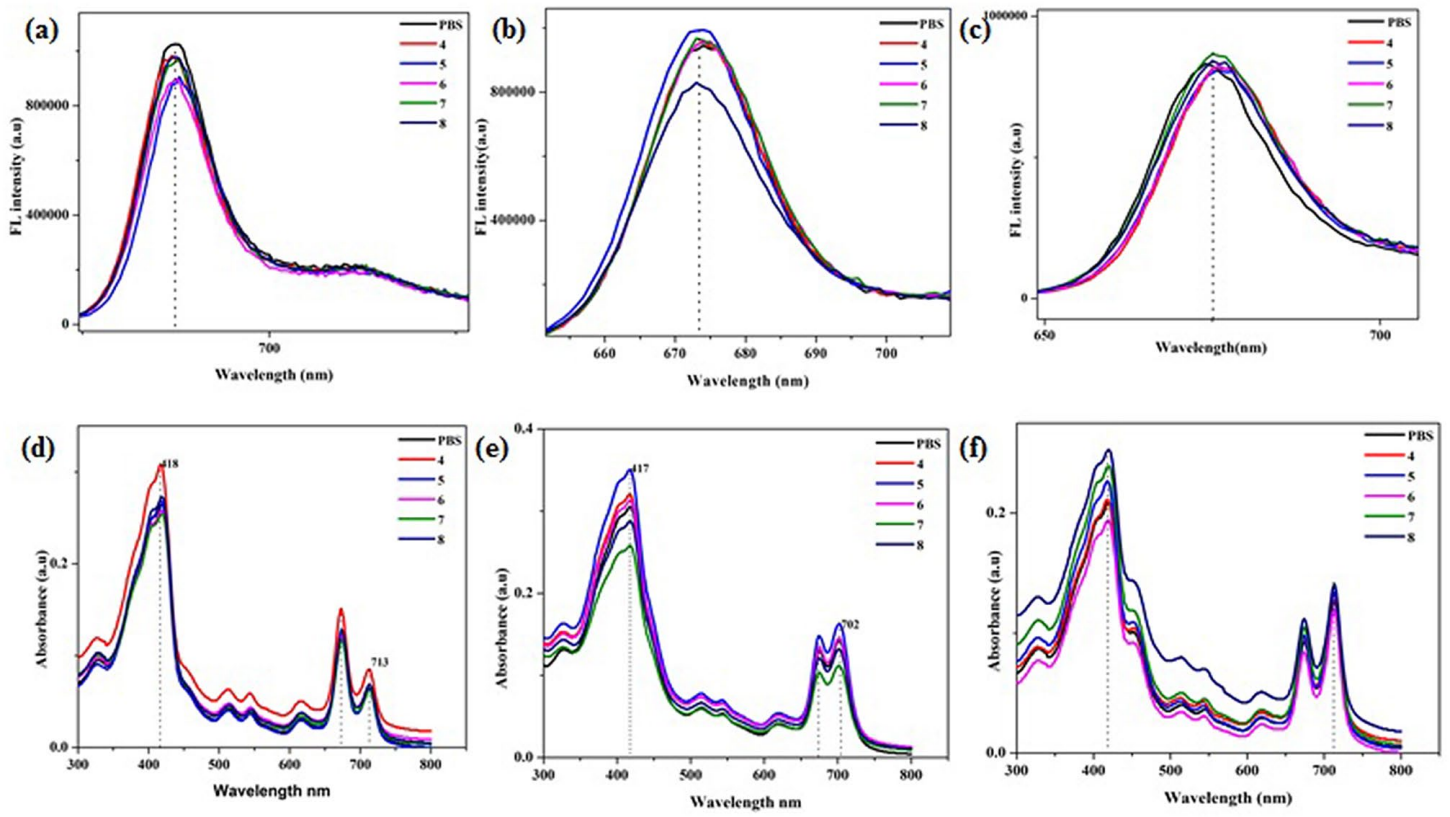
PL Spectra of conjugates at various pH values (**a**) PPa-PD1, (**b**) PPa-PD2, (**c**) PPa-PD3: UV–Vis spectra in different pH solutions (**e**) PPa-PD1, (**f**) PPa-PD2, (**g**) PPa-PD3.

As Xia et al. reported, the synthesized R-CPDs possess pH sensitivity which is an important characteristic for its application is biomedicines. PPa-PD hybrids were supposed to exhibit pH sensitivity. To obtain proof for this assumption, the effects of a broad range of pH from 4 to 8 on the optical properties of the nanohybrids were investigated. As shown in Fig. 8, the PD-hybrids displayed strong fluorescence signals at 674 nm in a neutral environment, and the peak was shifted to 677 nm in a solution of 4 pH (Fig. 8b). The FL intensity showed a change with pH in a range of 5 to 6 in case of π–π stacking which was consistent with the results of Xia et al. While FL intensity decreased at 8 pH when p-dots were covalently bonded with PPa might be due to the deprotonation of the porphyrin in an alkaline environment. pH sensitive response was evaluated using UV–vis absorption spectra. Figure 8d–f showed the absorbance’s of pdot-hybrids from pH 4 to 8. The peak at 417 and 702 (713) nm decreased in the pH solution of 7 while it increased in acidic solution (PPa-PD1, PPa-PD2). A slight shift of 2–3 nm was observed in each drug solution at 417 nm in basic solutions. Therefore, the pH-responsive nanoprobe based on PDs might be suitable for pH sensing.

## Conclusion

In short, we synthesized red emission carbon based polymers dots and later incorporated them with a widely used photosensitizer PPa through π–π stacking and covalent bonding. We also developed one pot facile synthesis of the hybrid drug. The synthesized hybrids were characterized by TEM, FT-IR, ^1^HNMR, UV–Vis and, fluorescence spectrophotometry. The absorbance of the PD conjugates was found to be increased which confers the successful conjugation. The resulting Pdots possessed excellent stability as well as yields high singlet oxygen. Facile one pot synthesis of the nanohybrids was achived at low temperature. The one pot synthesis was an efficient method to synthesize the conjugate as this method not only reduced the synthesis time but also enhanced the PDT efficacy of the hybrid. DLS results propose that the temperature has effect on the aggregation of the conjugates.The cytotoxicity and photodynamic effects of the Pdots were evaluated in Eca-109 cells by MTT assay. The dark toxicity of the photosensitizer dope p-dots was reduced as compared to pyropheophorbide-α, indicating that the hybrid drugs could efficiently damage cancer cells due to the high-yield ^1^O_2_ generation. Accordingly, PPa-PDs could be accumulated in Eca-109 cells, leading to the remarkable photodynamic therapeutic effect under laser irradiation. The hemolysis ratio of nanohybrids suggested that the synthesized composite drugs were biocompatible. The nano-composites displayed a change in fluorescence signal in different pH solutions suggests that the conjugate inherit the pH sensitivity of the PDs. The synthesized nanohybrids possessed good solubility, high ROS generation and low dark toxicity. This study will offer a new perspective in the design of hybrid photosensitizers and may advance biomedical research using such biomaterials and nanomedicine.

## Supplementary Information

Supplementary Information.
