# Supplementary material for: Synthesis and in vitro PDT evaluation of red emission polymer dots (R-CPDs) and pyropheophorbide-α conjugates
Source: Sci Rep. 2021 May 11;11:10013. doi: 10.1038/s41598-021-89081-y (PMC8113465; doi:10.1038/s41598-021-89081-y)
Supplement: Supplementary file 1 — Supplementary Information. [file 41598_2021_89081_MOESM1_ESM.docx]

**Synthesis and *invitro* PDT evaluation of Red emission polymer dots (R-CPDs) and pyropheophorbide-α conjugates**

Faiza sajjad^a^, Yi-Jia Yan^b^*, Davor Margetić^c^*, Zhi-Long Chen ^a^*

^a^ Department of pharmaceutical Science & Technology, College of Chemistry and Biology, DongHua University, Shanghai 201620, China

^b^ Shanghai Xianhui Pharmaceutical Co., Ltd, Shanghai, 200433, China

^c^Division of Organic Chemistry and Biochemistry, RuđerBošković Institute, Bijenička c. 54, 10000, Croatia

Faiza Sajjad: [fsajjad37@yahoo.com](mailto:fsajjad37@yahoo.com)

*Corresponding author: Yi-Jia Yan. E-mail: [y_yan11@sina.com](mailto:y_yan11@sina.com)

Davor Margetić, E-mail: [margetid@irb.hr](mailto:margetid@irb.hr)

Zhi-Long Chen. E-mail: zlchen1967@qq.com

**Supporting Data**

**Fig S1a. ^1^HNMR of PPa-PD1 in DMSO**

**Fig S1b. ^1^HNMR of PPa-PD2 in DMSO**

**Fig S1c. 1HNMR of PPa-PD3 in DMSO**


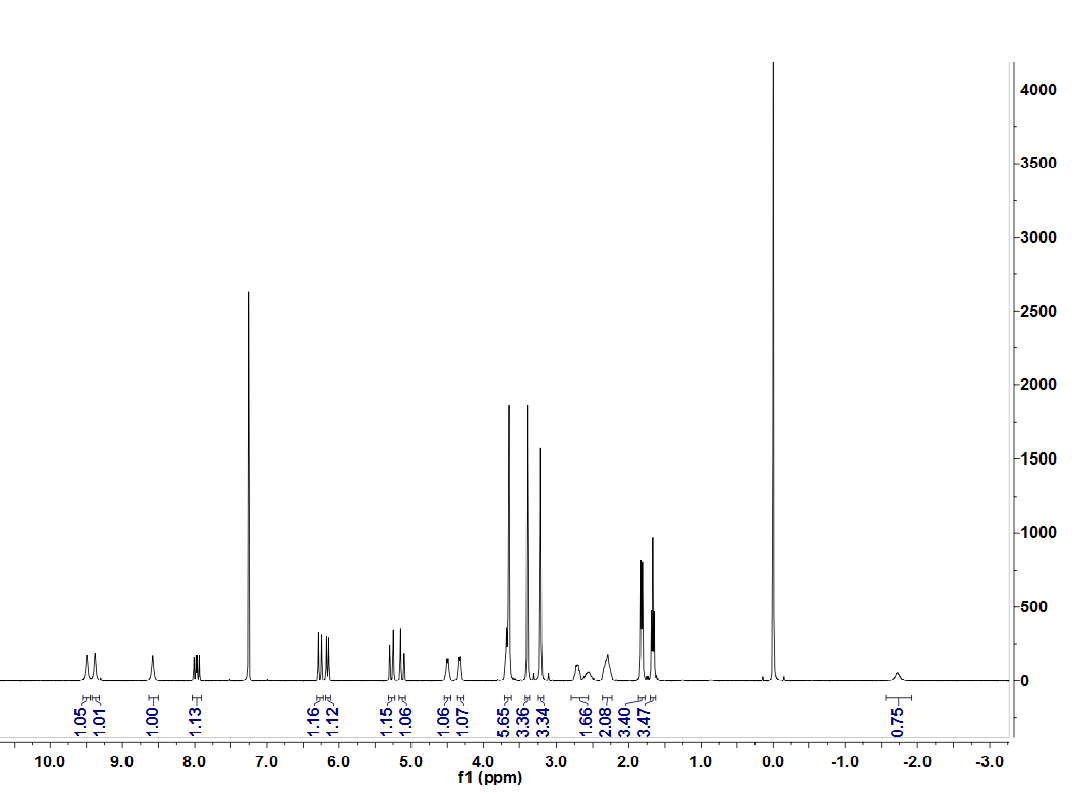


**Fig S1d.** **^1^HNMR of PPa in CDCl_3_**


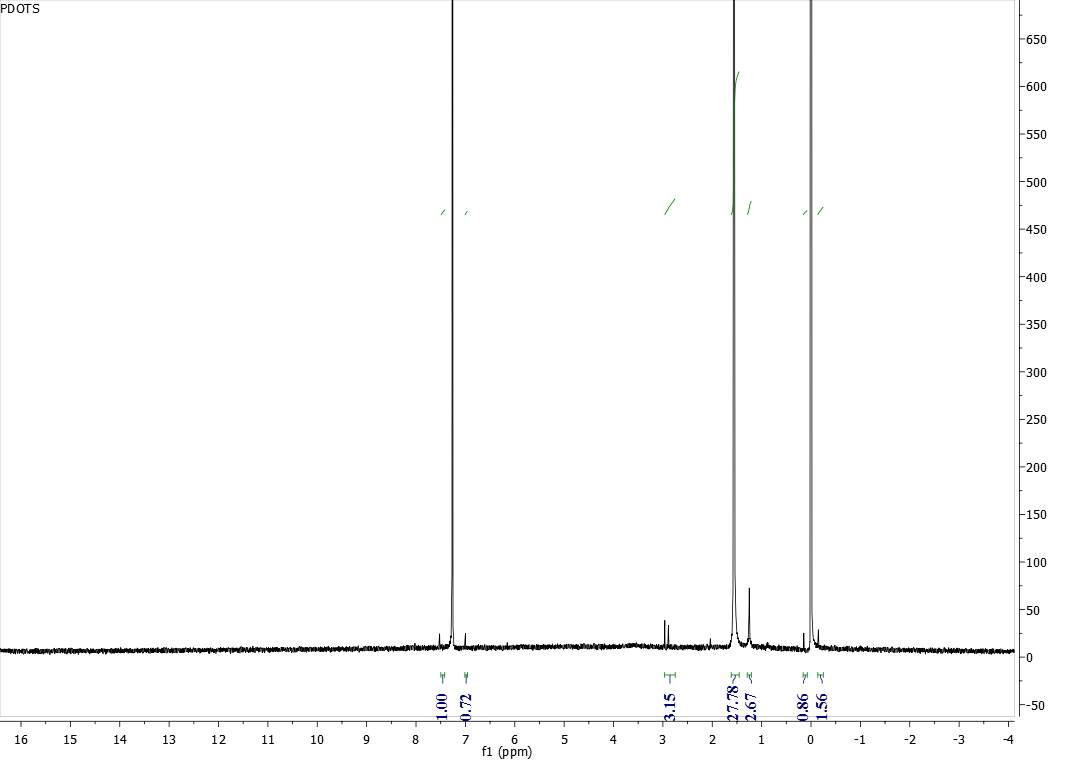


**S1e. ^1^HNMR of Pdots in CDCl3**


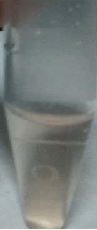


**PPa-PD1**


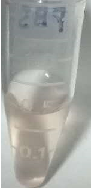


**PBS**


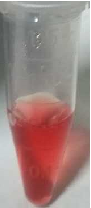


**H_2_O**


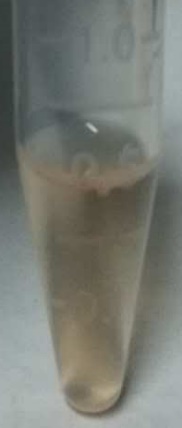


**PPa**


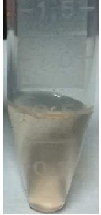


**PPa-PD3**


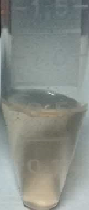


**PPa-PD2**

**Fig S2a. Hemolytic effect of PPa-PD conjugates**


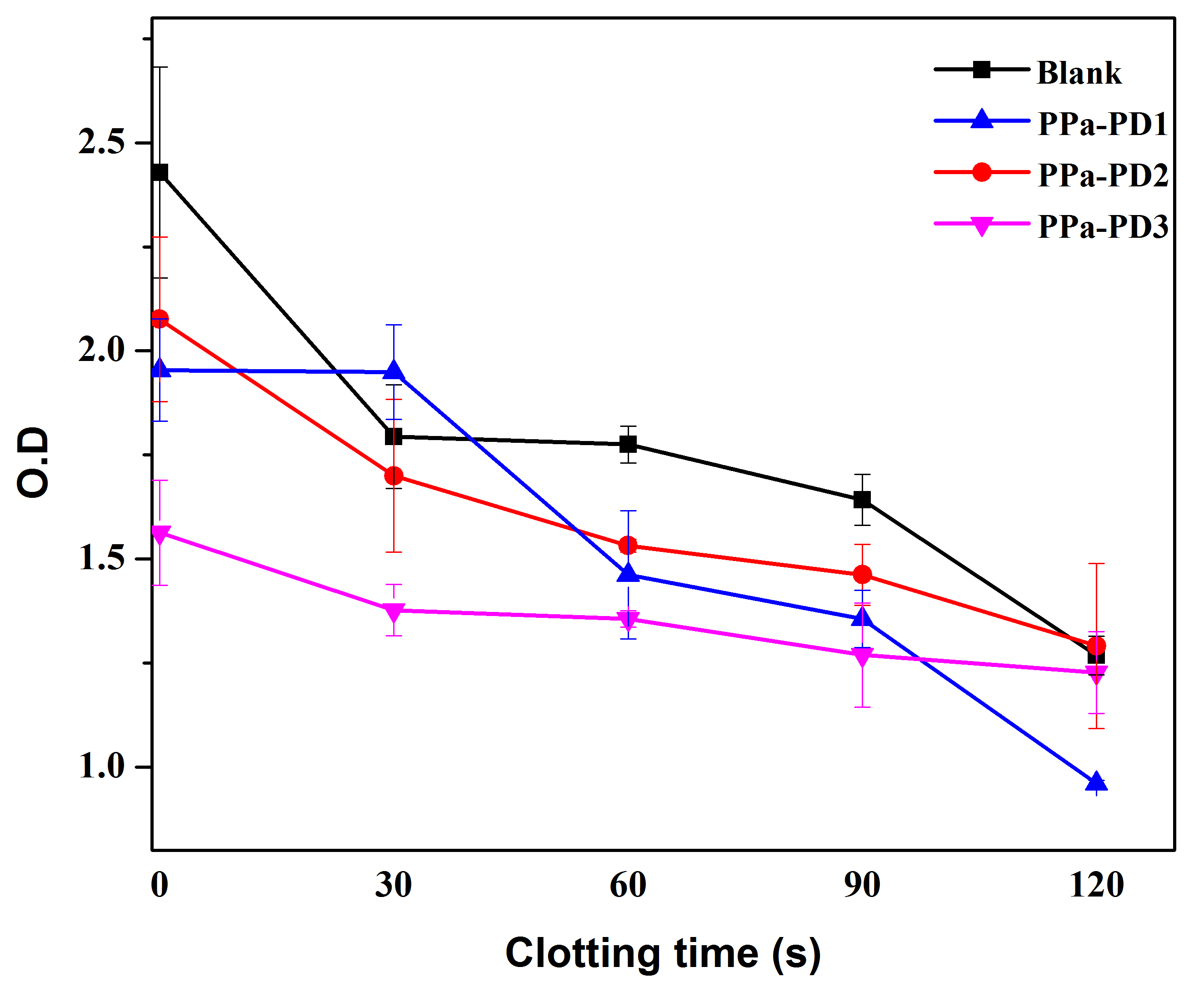


**Fig S2b.** The effect of drugs on blood clotting
